# The effect of CFTR modulators on a cystic fibrosis patient presenting with recurrent pancreatitis in the absence of respiratory symptoms: a case report

**DOI:** 10.1186/s12876-019-1044-7

**Published:** 2019-07-11

**Authors:** J. Dixon Johns, Steven M. Rowe

**Affiliations:** Division of Pulmonary, Allergy and Critical Care Medicine, University of Alabama at Birmingham, 1918 University Blvd., MCLM 706, Birmingham, AL 35294 USA

**Keywords:** Recurrent pancreatitis, Cystic fibrosis, CFTR, Ivacaftor, Pancrelipase

## Abstract

**Background:**

Cystic fibrosis (CF) is a genetic disorder of the epithelial CFTR apical chloride channel resulting in multi-organ manifestations, including pancreatic exocrine secretion. In the pancreas, CFTR abnormality results in abnormally viscous secretions that obstruct proximal ducts leading to fibrotic injury and ultimately pancreatic insufficiency in 85% of the CF population. CFTR modulators, including the potentiator ivacaftor, augment channel gating to restore 30–50% of CFTR-mediated anion transport. While CFTR modulation has been shown to alkalinize the pH of the alimentary tract and potentially augment pancreatic enzyme activity, the effect of ivacaftor on recurrent pancreatitis is emerging. Here we describe a case of a patient with CF (*R117H/7 T/F508del*) who presented with recurrent pancreatitis who was effectively treated with ivacaftor in the absence of respiratory symptoms.

**Case presentation:**

A 24-year-old white male with past medical history of recurrent acute pancreatitis presented for evaluation following a referral from an outside hospital. The patient reported a lifetime of gastrointestinal symptoms requiring over 20 hospitalizations for pancreatitis in the last 10 years. Prior U/S and CT imaging for pancreatitis ruled out gallstones or anatomical etiologies. Family history included a brother with CF carrier status who suffered from recurrent acute pancreatitis. Sweat chloride testing was suggestive of CFTR dysfunction (57 mmol/L). Genetic testing demonstrated disease causing *CFTR* mutations: *R1117H/7 T/F508del*. Patient was prescribed pancrelipase, however, he reported worsened gas and diarrhea symptoms. Pancrelipase was discontinued and the patient was prescribed ivacaftor 150 mg BID. After 6 weeks of ivacaftor treatment, patient reported improved gastrointestinal symptoms. For an additional 19 months, patient reported no episodes of pancreatitis until he discontinued ivacaftor. Over the next 3 weeks, patient experienced progressive nausea and sharp epigastric pain and laboratory studies confirmed pancreatitis. Patient was subsequently lost to follow up.

**Conclusions:**

These findings support a possible relationship between the use of CFTR modulators, such as ivacaftor, in the management of recurrent pancreatitis in the setting of patients with cystic fibrosis and a *CFTR* mutation with residual CFTR activity or otherwise known to be responsive in vitro. Ivacaftor may be useful for recurrent pancreatitis, even in the absence of respiratory morbidity.

## Background

Cystic fibrosis (CF) is a genetic disorder of the epithelial CFTR apical chloride channel resulting in multi-organ manifestations, including pancreatic exocrine secretion. In the pancreas, CFTR abnormality results in abnormally viscous secretions that obstruct proximal ducts leading to fibrotic injury and ultimately pancreatic insufficiency in 85% of the CF population [1]. There is a relatively strong correlation between CFTR genotype and pancreatic phenotype. While severe CFTR genotypes are associated with early-onset pancreatic insufficiency and severe respiratory disease [2], genotypes with one or more residual CFTR function mutation, such as *R117H CFTR*, can initially present with preserved respiratory function and episodes of recurrent, acute pancreatitis without exocrine insufficiency [3]. CFTR potentiators, including ivacaftor, augment channel gating to restore 30–50% of CFTR-mediated anion transport in patients with gating and residual function mutations, like R117H. While CFTR modulation has been shown to alkalinize the pH of the alimentary tract and potentially augment pancreatic enzyme activity, the effect of ivacaftor on recurrent pancreatitis is emerging. Here we describe a case of a patient with CF (*R117H/7 T/F508del*) who presented with recurrent pancreatitis who was effectively treated with ivacaftor in the absence of respiratory symptoms.

## Case presentation

A 24-year-old white male with past medical history of recurrent acute pancreatitis, gastroesophageal reflux disease, and obesity presented for evaluation following a referral from an outside hospital after a 3-week hospitalization due to refractory acute pancreatitis. The patient reported gastrointestinal symptoms including colicky discomfort and reflux as a teenager that evolved into sharp, severe abdominal pain requiring over 20 hospitalizations for pancreatitis in the last 10 years. Hospitalizations for recurrent acute pancreatitis ranged in severity from multi-day hospitalizations for pain management, anti-emetic therapy, bowel rest, and supportive care to one- and two-day hospitalizations where symptoms severity was less intense; less severe episodes that did not require hospitalization were also increasingly common. With the initial episodes, alcohol use increased the likelihood of symptoms occurring, but complete abstinence for the last two years did not alter the likelihood of recurrence. While episodes occurred initially every 6–12 months, the frequency increased over recent years to approximately every 3 months. He denied episodes correlated to diet, but alcohol use did increase the frequency of pancreatitis events. Prior ultrasound and computed tomography imaging for pancreatitis ruled out gallstones or anatomical etiologies. The patient was treated with proton-pump inhibitors and avoided spicy foods and alcohol with minimal resolution of symptom severity or frequency.

On presentation, the patient reported intermittent right upper quadrant pain with nausea and episodes of constipation and diarrhea, but without evidence of fat malabsorption. He denied emesis, fever, or weight loss, and also denied sinus or respiratory symptoms. He had no current alcohol use. Family history included a brother with CF carrier status who suffered from recurrent acute pancreatitis.

Physical examination was significant for obesity (BMI = 39.4 kg/m^2^), mild right upper quadrant tenderness, and negative pulmonary findings. Laboratory evaluation was notable for normal LFTs with mildly elevated lipase (116 U/L). Despite obesity, he had severely diminished fecal elastase (< 15.0 μg/g feces) and decreased Vitamin D (11.7 ng/mL), suggesting at least intermittent or evolving pancreatic insufficiency. Computed tomography of abdomen demonstrated acute interstitial edematous pancreatitis without evidence of necrosis and thickening of duodenum likely due to pancreatic inflammation. Abdominal ultrasound showed hepatomegaly with hepatic steatosis with abnormal flow within hepatic veins suggesting possible hepatic fibrosis. Pulmonary function testing resulted in 87 and 90% of predicted FVC and FEV_1_, respectively, with a preserved FEV_1_/FVC (0.80) High-resolution computed tomography of the chest showed modest air trapping in the dependent lungs with early signs of bronchial wall thickening; sputum cultures were negative for CF pathogens. Sweat chloride testing indicated CFTR dysfunction (57 mmol/L). Genetic testing demonstrated disease causing *CFTR* mutations: *R1117H/7 T/F508del*.

**Fig. 1:**
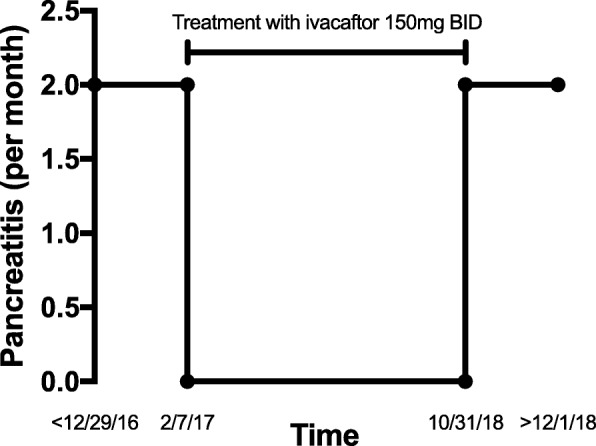
Pancreatitis Episodes (per month). Representative figure demonstrating the pancreatitis rate (per month) from 12/29/16 through 12/1/18 with line indicating treatment with ivacaftor 150 mg

Awaiting genotyping, the patient was prescribed pantoprazole 40 mg daily and pancrelipase 20,880 USP units TID with meals, and he declined other respiratory interventions. On follow-up 6 weeks later, he reported that low dose pancrelipase worsened gas and diarrhea symptoms. Pancrelipase was discontinued and the patient was prescribed ivacaftor 150 mg BID. After 6 weeks of ivacaftor treatment, patient reported improved gastrointestinal symptoms. For an additional 19 months, patient reported no episodes of pancreatitis, representing a marked improvement in frequency (Fig. 1), until he discontinued ivacaftor. Over the next 3 weeks, patient experienced progressive nausea and sharp epigastric pain and laboratory studies confirmed pancreatitis with elevated amylase (231) and lipase (1221), but normal LFTs. Patient experienced 2 episodes of pancreatitis in the first month off medication. They were instructed to resume ivacaftor but subsequently lost to follow up.

## Discussion and conclusion

Mild class IV (R117H, etc.) and V (3849 + 10kbC, etc.) CFTR mutations result in decreased channel conductance and reduced synthesis, respectively. In the pancreas, this results in preserved acinar and ductular function compared with severe class I, II, or III mutations [4]. Although initially pancreatic sufficient, residual function mutations demonstrate a high propensity for ductal blockage and inflammation, resulting in pancreatitis [5]. Over time, these episodes of recurrent pancreatitis develop defective acinar pancreatic secretion and a decline in pancreatic exocrine function, with 20% becoming pancreatic insufficient [6]. R117H is particularly complex exon 4 missense mutation, as it is affects exon 9 splicing and is impacted by the status of the Poly-T tract (5 T, 7 T, or 9 T variants) on intron 8. Poly-T tract 5 T variant is associated with increased rates of male infertility caused by congenital bilateral atresia of vas deferens (CBAVD), bronchiectasis, and chronic idiopathic pancreatitis [7]. The 7 T variant also possesses clinical significance in relation to R1117H, with patients typically presenting with CBAVD with late-onset respiratory disease [8].

Treatment of CFTR dysfunction has gained significant momentum with the advent of CFTR modulators such as ivacaftor. Recent studies have shown that ivacaftor improved FEV_1_, reduced pulmonary exacerbations, and reduced events leading to hospitalizations in select patients [9, 10] , including those with R117H, regardless of 5 T/7 T status. However, its effects on the gastrointestinal tract are less well understood. Recent data suggests ivacaftor augments gastrointestinal physiology, with increased bicarbonate secretion and increased weight gain thought to be related to improved PERT activity [11]; however, this effect could also be related to partial rescue of pancreatic bicarbonate secretion via rescue of a proportion of intact pancreatic ducts. This is further suggested by the partial rescue of pancreatic exocrine function measured by increased fecal elastase levels in children with G551D were treated with ivacaftor, where 20–25 and 67% exhibited FE levels consistent with pancreatic sufficiency (> 200 μg/g feces), in age 2–5 and 1–2, respectively [12, 13]. While organ preservation in young CF patients including the pancreas seems increasingly likely, the same may be true for selected adults with preserved organ function, if instituted prior to irreversible injury. Tezacaftor, a corrector of F508del function, in combination with ivacaftor augmented FE levels in patients age 12 and above with F508del and a residual function mutation [14]; this study may be the most relevant to the case presented here. Similarly, resolution of recurrent pancreatitis may also be possible. Carrion et al. reported a case series detailing a retrospective study of 6 patients with CF and a history of pancreatitis (between 1 to 5 episodes in the prior 12 months) due to various mutations (*F508del/R117H/5 T, F508del/R117H/7 T, F508del/S1255P, F508del/G551S, F508del/D1152H,* and *F508del*/unknown thymidine tract length) between ages 11.5 to 60 years old. After administration of ivacaftor, none of the patients had recurrence of pancreatitis or hospitalization due to gastrointestinal morbidity in the following 3 to 12 months following treatment [15]. This case adds to this experience, and further demonstrates causality given pancreatitis recurred when ivacaftor was abruptly discontinued, a unique experience.

As with any chronic therapy, care must be considered when initiating patients on ivacaftor therapy. While generally well tolerated in patients with CF [16], there is little published experience in patients with CFTR dysfunction from other non-genetic causes [17], although diarrhea is a theoretical risk due to the contribution of CFTR fluid secretion in the bowel [18]. While cough, headache, and URI are common with ivacaftor use, these are likely associated with underlying CF. Elevated liver enzymes have occurred, and monitoring liver function tests quarterly for the first year of use is warranted to detect drug related hepatic injury; should elevated tests remain persistent, ivacaftor should be held, noting this finding could also be due to underlying CF, concomitant medical therapy, and underlying hepatic obstruction in patients at risk for pancreatitis. Ivacaftor is metabolized by CYP3A4, thus inducers and inhibitors of CYP3A4 should be used with caution and appropriate dose adjustment [19].

Overall, this case supports a possible relationship between the use of CFTR modulators, such as ivacaftor, in the management of recurrent pancreatitis in the setting of patients with cystic fibrosis and a *CFTR* mutation with residual CFTR activity or otherwise known to be responsive in vitro. Ivacaftor may be useful for recurrent pancreatitis, even in the absence of respiratory morbidity.

## Data Availability

The datasets used and/or analyzed during the current study are available from the corresponding author on reasonable request.

## Abbreviations

BID: Bis in die (twice a day)
BMI: Body mass index
CBAVD: Congenital bilateral atresia of vas deferens
CF: Cystic fibrosis
CFTR: Cystic fibrosis transmembrane conductance regulator
FE: Fecal elastase
FEV_1_: Forced expiratory volume
FVC: Forced vital capacity
LFT: Liver function tests
PERT: Pancreatic enzyme replacement therapy
TID: Ter in die (thrice a day)
USP units: United States pharmacopeia units

## References

[1] Nousia-Arvanitakis S (1999). Cystic fibrosis and the pancreas: recent scientific advances. J Clin Gastroenterol.

[2] Augarten A (2008). The changing face of the exocrine pancreas in cystic fibrosis: the correlation between pancreatic status, pancreatitis and cystic fibrosis genotype. Eur J Gastroenterol Hepatol.

[3] Ooi CY (2011). Type of CFTR mutation determines risk of pancreatitis in patients with cystic fibrosis. Gastroenterology.

[4] Sharer N (1998). Mutations of the cystic fibrosis gene in patients with chronic pancreatitis. N Engl J Med.

[5] Ahmed N (2003). Molecular consequences of cystic fibrosis transmembrane regulator (CFTR) gene mutations in the exocrine pancreas. Gut.

[6] Couper RT (1992). Decline of exocrine pancreatic function in cystic fibrosis patients with pancreatic sufficiency. Pediatr Res.

[7] Sebastian S (2004). Multicenter characterization and validation of the intron-8 poly(T) tract (IVS8-T) status in 25 Coriell cell repository cystic fibrosis reference cell lines for cystic fibrosis transmembrane conductance regulator (CFTR) gene mutation assays. Clin Chem.

[8] Kiesewetter S (1993). A mutation in CFTR produces different phenotypes depending on chromosomal background. Nat Genet.

[9] Wainwright CE, Elborn JS, Ramsey BW (2015). Lumacaftor-Ivacaftor in patients with cystic fibrosis homozygous for Phe508del CFTR. N Engl J Med.

[10] Cholon DM, Esther CR, Gentzsch M (2016). Efficacy of lumacaftor-ivacaftor for the treatment of cystic fibrosis patients homozygous for the F508del-CFTR mutation. Expert Rev Precis Med Drug Dev.

[11] Gelfond D (2017). Impact of CFTR modulation on intestinal pH, motility, and clinical outcomes in patients with cystic fibrosis and the G551D mutation. Clin Transl Gastroenterol.

[12] Davies JC (2016). Safety, pharmacokinetics, and pharmacodynamics of ivacaftor in patients aged 2-5 years with cystic fibrosis and a CFTR gating mutation (KIWI): an open-label, single-arm study. Lancet Respir Med.

[13] Rosenfeld M (2018). Ivacaftor treatment of cystic fibrosis in children aged 12 to <24 months and with a CFTR gating mutation (ARRIVAL): a phase 3 single-arm study. Lancet Respir Med.

[14] Rowe SM (2017). Tezacaftor-Ivacaftor in residual-function heterozygotes with cystic fibrosis. N Engl J Med.

[15] Carrion A (2018). Reduction of recurrence risk of pancreatitis in cystic fibrosis with Ivacaftor: case series. J Pediatr Gastroenterol Nutr.

[16] Habib AR (2019). A systematic review of the clinical efficacy and safety of CFTR modulators in cystic fibrosis. Sci Rep.

[17] Solomon GM (2016). Pilot evaluation of ivacaftor for chronic bronchitis. Lancet Respir Med.

[18] Lambert JA (2014). Cystic fibrosis transmembrane conductance regulator activation by roflumilast contributes to therapeutic benefit in chronic bronchitis. Am J Respir Cell Mol Biol.

[19] Ivacaftor (Kalydeco) for cystic fibrosis. Med Lett Drugs Ther, 2012. 54(1388): p. 29–30. 22499233

